# Organizational Culture Shock and Knowledge Transfer Behavior Among Newly Hired Engineering PhDs in Firms: A Self-Determination Perspective

**DOI:** 10.3390/bs16050752

**Published:** 2026-05-12

**Authors:** Yang Zou, Qiqi Li, Wenjing Yuan, Xianwei Liu

**Affiliations:** 1 College of Business Administration, Capital University of Economics and Business, Beijing 100070, China; zouyang@cueb.edu.cn; 2 School of Economics and Management, Beijing Forestry University, Beijing 100083, China; lqq205313@bjfu.edu.cn; 3 Career Development Center, Beihang University, Beijing 100191, China; 09225@buaa.edu.cn; 4 School of Public Administration, Beihang University, Beijing 100191, China; 5 Research Center for Reform and Development of Graduate Education, Beihang University, Beijing 100191, China

**Keywords:** organizational culture shock, autonomous motivation, controlled motivation, amotivation, knowledge transfer behavior, engineering PhDs in firms, self-determination theory

## Abstract

Engineering PhDs are expected to act as key knowledge brokers between universities and firms. Drawing on self-determination theory (SDT), we examined the associations among organizational culture shock (OCS), SDT motivations, and knowledge transfer behavior (KTB) of newly hired engineering PhDs in Chinese firms. We also explored whether these associations varied across subgroups defined by gender, career goal at PhD entry, prior industry collaboration experience, and dissertation orientation. Data were collected from 466 engineering PhDs within one year after they entered firms. Structural equation modeling (SEM) analysis revealed that OCS was negatively associated with KTB, including indirect associations through the three types of SDT motivations. Autonomous motivation was positively associated with KTB, whereas controlled motivation and amotivation were negatively associated with it. Multi-group SEM analyses further indicated that the strength of the structural pathways varied across subgroups defined by gender, career goal at PhD entry, industry collaboration experience, and dissertation orientation. These findings suggest that OCS may represent a micro-level barrier to university–industry knowledge transfer. They also indicate that firms and universities may help support knowledge transfer by facilitating PhDs’ adjustment and autonomous motivation.

## 1. Introduction

Over the past three decades, shifts in the academic labor market and firms’ rising demand for advanced human capital have steered an increasing share of PhD graduates, particularly in science, technology, engineering, and mathematics (STEM), toward industry employment (Bloch et al., 2015; Zheng et al., 2024). Engineering doctorates constitute the largest doctoral cohort worldwide and are closely aligned with firms’ research and development (R&D) needs. For instance, in the United States, the proportion of engineering PhDs employed in industry rose from 64.6% in 1994 to 74.2% in 2024 (National Science Foundation, 2024), and leading Chinese engineering universities report industry placements above 50% (Luo et al., 2022). Beyond technical expertise and research training, firms increasingly view PhDs as “knowledge brokers” who connect academic science with industrial innovation (Garcia-Morante et al., 2025). This expectation reflects firms’ strategic interest in recruiting PhDs who can bring doctoral knowledge into industrial practice and support innovation-oriented work.

When embedded in industrial R&D environments, PhDs can channel frontier knowledge into process and product development, thereby reducing uncertainty and costs (Herrera & Nieto, 2016) and expanding firms’ absorptive capacity through their academic networks (Thune, 2010). In line with this promise, China and other countries with well-established doctoral education systems have advanced policy agendas to strengthen university–industry knowledge flows through doctoral training reforms (e.g., Borrell-Damian et al., 2010; Kitagawa, 2014; Liu et al., 2020). Yet, while prior work documents macro-level employment trends (e.g., Auriol et al., 2013) and meso-level factors influencing firms’ PhD recruitment (e.g., Herrera & Nieto, 2015), far less is known about the micro-level experiences of PhDs once they enter firms (Germain-Alamartine et al., 2021), especially the challenges they face and how those challenges shape their knowledge transfer behavior (KTB) (Galimberti, 2023). Following Tho (2017), KTB concerns the acquisition or internalization of knowledge and, critically, its application in the workplace; in the present study, we use KTB in this application-focused sense, referring specifically to newly hired PhDs’ use of doctoral-acquired knowledge, skills, methods, techniques, and analytical capabilities in their current firm work. More broadly, knowledge transfer becomes meaningful when knowledge moves across contexts and contributes to work-related action and performance (Argote & Ingram, 2000).

Organizational culture shapes the routines through which knowledge moves and thus determines the feasibility of transfer (Rahman et al., 2018). Greater cultural distance between knowledge source and recipient increases coordination and translation costs, thereby hindering transfer (Fong Boh et al., 2013). For PhDs transitioning from academia to industry, such incongruence is often acute, given the sharp differences in organizational structures and knowledge-production logics (Leydesdorff & Meyer, 2006). New PhD hires may therefore encounter organizational culture shock (OCS), namely, a disorienting misfit of values, expectations, and work practices during the transition from academic to industrial settings (Skakni et al., 2022). Early career stages are crucial for this adaptation. The “entry-job hypothesis” suggests that adjustment during the initial employment period has long-term implications for PhDs’ professional success (Enders, 2002).

Knowledge transfer is not an automatic process. It depends on the willingness of potential gatekeepers to engage (Polanyi, 2015). While engineering PhDs generally possess the technical expertise for knowledge transfer, their motivation significantly affects the extent, quality, and creativity of their engagement (Arzenšek et al., 2014). Self-determination theory (SDT) is useful for explaining this process because it distinguishes among autonomous motivation, controlled motivation, and amotivation according to the degree of self-determination (Ryan & Deci, 2000). SDT also argues that contextual factors shape behavior by supporting or thwarting the basic psychological needs for autonomy, competence, and relatedness (Ryan & Deci, 2017). Applied to this study, OCS may frustrate these needs, thereby weakening autonomous motivation and strengthening controlled motivation or amotivation, which in turn may affect PhDs’ engagement in KTB (Sun et al., 2025). Moreover, individual characteristics such as gender (e.g., Marini, 2022), career goals at PhD entry (e.g., Kim et al., 2018), prior industry collaboration experience (e.g., Boman et al., 2026), and dissertation orientation (e.g., Roach & Sauermann, 2017) may shape how PhDs respond to organizational transition.

Despite these insights, two gaps remain. First, studies of PhDs in industry have focused mainly on employment destinations (e.g., Skakni et al., 2026), firms’ recruitment of doctoral graduates (e.g., Muurlink et al., 2024), and broad job satisfaction (Sala-Bubaré et al., 2024), while giving less attention to how early organizational experiences shape PhDs’ actual workplace behaviors after they enter firms. This gap is particularly important for knowledge transfer, because PhDs’ value to firms depends not only on their advanced training but also on whether they can transfer doctoral knowledge into industrial practice. Second, although SDT has recently been applied to doctoral career research, existing studies have mainly used it to explain doctoral students’ career preferences and choices (e.g., Meuleners et al., 2023). Less is known about how SDT can explain the motivational process through which post-graduation organizational experiences influence PhDs’ workplace behaviors. Addressing these gaps can clarify whether OCS is associated with KTB, through which motivational mechanisms it operates, and under what individual conditions.

Guided by SDT, this study develops and tests an integrative framework linking OCS to KTB through motivational mechanisms among newly hired engineering PhDs in firms, while considering how individual characteristics and prior experiences differentiate these relationships. Specifically, this study addresses three research questions:

RQ1: How is OCS associated with KTB among newly hired engineering PhDs in firms?
RQ2: Do autonomous motivation, controlled motivation, and amotivation mediate the relationship between OCS and KTB?
RQ3: Do the structural relationships among OCS, SDT motivations, and KTB differ across subgroups defined by gender, career goal at PhD entry, prior industry collaboration experience, and dissertation orientation?

By addressing these questions, this study makes three contributions. First, it extends research on PhDs’ transition to industry by shifting attention from employment destinations and firm recruitment to the behavioral consequences of early organizational experiences. Second, it contributes to the knowledge transfer literature by showing how different qualities of motivation help explain the association between OCS and KTB, rather than treating motivation as a single general factor. Third, it extends SDT to the context of university–industry knowledge transfer by examining newly hired engineering PhDs as knowledge brokers and by identifying individual characteristics and prior experiences as boundary conditions of the OCS–motivation–KTB process. These contributions also offer implications for doctoral education policy and corporate human resource practices aimed at supporting PhDs’ transition into industry.

## 2. Theoretical Background and Hypothesis Development

### 2.1. Self-Determination Theory

Work motivation shapes the nature, direction, intensity, and persistence of behaviors associated with job performance (Brown, 2007). SDT, developed by Deci and Ryan, is one of the most influential frameworks for explaining workplace motivation (Deci & Ryan, 1985). At its core, SDT distinguishes between amotivation and motivation. Amotivation denotes the absence of intentionality, often arising when individuals do not value an activity, feel incompetent, or fail to perceive a link between effort and outcomes. Controlled motivation includes external regulation and introjected regulation and reflects actions driven by external contingencies or partially internalized pressures, such as working to obtain rewards, avoid punishments, or reduce guilt. Autonomous motivation encompasses intrinsic motivation, that is, engaging in an activity out of genuine interest or enjoyment, as well as well-internalized forms of extrinsic motivation (identified and integrated regulation), where behavior is aligned with personal values and a coherent sense of self. Individuals with autonomous motivation experience volition and psychological freedom in their work (Ryan & Deci, 2000).

These distinctions are especially relevant in organizational contexts because motivational quality influences performance, persistence, and well-being of employees (Manganelli et al., 2018). SDT further explains how environmental conditions shape motivation and behavior. When the basic psychological needs for autonomy, competence, and relatedness are satisfied, individuals are more likely to experience willingness, volition, and choice in work activities (Ryan & Deci, 2017). Such need satisfaction fosters autonomous motivation and supports higher-quality performance (Gagné, 2009). Conversely, when these needs are frustrated, individuals may rely more on controlled motivation or become amotivated, which can weaken engagement and effectiveness.

This framework is well suited to the present study because OCS represents a contextual challenge that may frustrate newly hired PhDs’ basic psychological needs. Misaligned values, unclear role expectations, and ambiguous organizational status may reduce their sense of autonomy, competence, and relatedness after entering firms. As a result, OCS may shift their motivation away from autonomous engagement and toward controlled or amotivation, thereby influencing their willingness to acquire, apply, and share knowledge in the workplace. This logic is consistent with knowledge management research showing that different types of motivation can lead to different knowledge transfer outcomes under different environmental conditions (e.g., Ferger & Rechberg, 2024; Thomas & Gupta, 2022).

**Figure 1:**
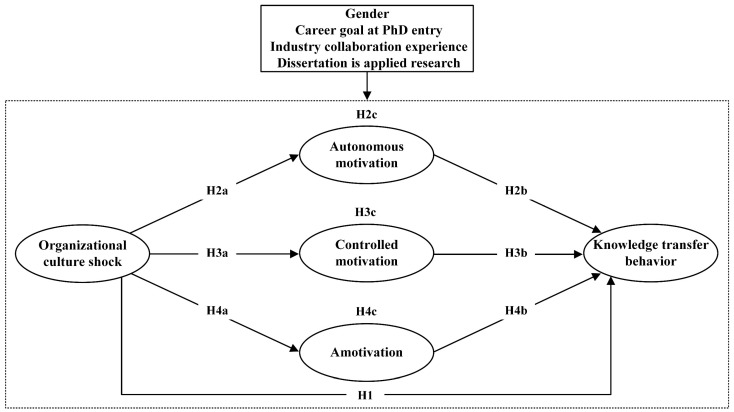
Theoretical model.

Building on SDT, this study examines autonomous motivation, controlled motivation, and amotivation as distinct mediating mechanisms linking OCS to KTB among newly hired engineering PhDs in firms. It also considers whether gender, career goals at PhD entry, prior industry collaboration experience, and dissertation orientation condition these relationships. Figure 1 presents the conceptual framework guiding this study.

### 2.2. Organizational Culture Shock and Knowledge Transfer Behavior

Organizational culture refers to the shared assumptions and interpretations of everyday practices that shape employees’ behavior (Alvesson, 2013). When PhD graduates transition from academia to industry, they often encounter OCS, a demanding process of adapting to unfamiliar routines, organizational values, and status hierarchies (Skakni et al., 2022). This challenge is especially salient for those who enter industry immediately after graduation and have limited prior work experience (Skakni et al., 2022). From the SDT perspective, these experiences can frustrate basic psychological needs for autonomy, competence, and relatedness (Ryan & Deci, 2017). Accordingly, we argue that OCS is likely to be negatively associated with PhDs’ KTB for the following reasons.

First, daily functioning in firms is characterized by more structured schedules, interdependent teamwork, and performance controls that contrast with the relative autonomy and flexibility of academic settings (Skakni et al., 2022). Such changes constrain autonomy, reduce opportunities for self-organization among knowledge brokers, and thereby dampen engagement in knowledge transfer (Hammami et al., 2013; Tho, 2017). Second, dominant industrial values, such as efficiency, pragmatism, and profitability, can conflict with academia’s emphasis on long-term intellectual exploration (H. Li & Horta, 2024). This value incongruence hinders the internalization of organizational goals and weakens discretionary behaviors that are central to effective knowledge transfer (Buch et al., 2015; De Grande et al., 2014). Third, hierarchical structures in firms can reduce PhDs’ perceived status and recognition compared with the relative autonomy and expert identity they previously enjoyed. Newcomers may perceive a loss of researcher status and diminished autonomy in non-academic roles (Skakni et al., 2022). This perceived erosion of status and autonomy may be linked to lower perceived competence and weakened social connectedness, thereby limiting the capacity to generate and disseminate knowledge across organizational boundaries (Hernando, 2015). In light of these arguments, we propose the following hypothesis:

**H1.**  *OCS is negatively associated with KTB among PhDs.*

### 2.3. Mediating Role of Autonomous Motivation

Within SDT, autonomous motivation denotes engaging in work with volition and personal endorsement (Ryan & Deci, 2000). Upon entry to firms, PhD graduates frequently encounter contexts that diverge from academic norms, which may render initial assignments less internalized and less congruent with personally endorsed goals (Skakni et al., 2022). SDT indicates that such contexts fail to support basic psychological needs and thereby erode autonomous regulation, reducing willingness to invest discretionary effort (Deci et al., 2017). Consistent with this view, evidence on PhDs’ transitions beyond academia links perceived misfit in routines and values to reduced volition and engagement in new roles (Garcia-Morante et al., 2025; Rönkkönen et al., 2025). Accordingly, in industrial settings, OCS is expected to be negatively associated with autonomous motivation among newly hired PhDs.

Autonomous motivation, in turn, is closely tied to discretionary KTB. When work is experienced as meaningful and self-concordant, individuals allocate effort to learning, initiating exchanges, and sharing expertise beyond formal requirements (Gagné, 2009; Gagné & Deci, 2005). In knowledge-intensive environments, job designs that afford discretion and meaning reliably predict higher-quality knowledge sharing (Foss et al., 2009), whereas motivation often operates as a binding constraint on whether capable individuals actually transfer knowledge (Siemsen et al., 2008). Network research further shows that structural centrality alone is insufficient, and autonomous motivation is a critical ingredient linking position and ability to realized knowledge-sharing (Reinholt et al., 2011). For newly hired PhDs, whose distinctive value often lies in brokering and translating scientific know-how, lower autonomous motivation therefore implies fewer voluntary exchanges and less initiative in translation and application, resulting in reduced frequency and quality of KTB. Taken together, OCS is expected to be negatively associated with autonomous motivation, which is in turn expected to be positively associated with PhDs’ KTB. Accordingly, we hypothesize:

**H2a.**  *OCS is negatively associated with autonomous motivation among PhDs.*

**H2b.**  *Autonomous motivation is positively associated with KTB among PhDs.*

**H2c.**  *Autonomous motivation mediates the relationship between OCS and KTB among PhDs.*

### 2.4. Mediating Role of Controlled Motivation

OCS reflects a salient mismatch between newcomers’ internalized norms and the host organization’s expectations (Skakni et al., 2022). Within SDT, such mismatch shifts regulation toward external and introjected regulation and increases reliance on external contingencies and self-imposed pressure, rather than volitional, value-endorsed regulation (Deci & Ryan, 2000). PhD entrants who experience OCS are therefore more likely to approach knowledge behaviors with a compliance orientation aimed at meeting targets and avoiding negative evaluation, rather than with personally endorsed motives to collaborate and to provide in-depth explanations (Gagné, 2009).

When OCS is associated with higher controlled motivation, knowledge transfer tends to be dampened through two mechanisms. First, an emphasis on extrinsic incentives can unintentionally create a knowledge-market dynamic. As organizations raise rewards to elicit sharing, knowledge brokers foresee escalating thresholds and shrinking marginal returns to additional effort, which encourages effort minimization (Minbaeva & Santangelo, 2018). Anticipating this trajectory, employees operating under controlled motivation scale back their investment and offer only minimum-viable contributions sufficient to qualify for rewards or avoid sanctions. Such transaction-oriented arrangements can also elevate refusal to share and reinforce hostile attitudes toward sharing (Husted et al., 2012). Second, controlled motivation steers behavior toward meeting externally specified criteria rather than engaging in joint sensemaking. Employees then prioritize routinized, easily codified outputs and avoid time-intensive explanation and contextualization that enable deeper learning (Sun et al., 2025). Substantial personal investment in building valuable knowledge may cultivate psychological ownership and territorial responses, which curtail willingness to open one’s knowledge domain to others (Huo et al., 2016; Pierce et al., 2001). Under external pressure, these restraints depress both the quantity and the depth of knowledge transfer (Husted et al., 2012). Accordingly, we hypothesize:

**H3a.**  *OCS is positively associated with controlled motivation among PhDs.*

**H3b.**  *Controlled motivation is negatively associated with KTB among PhDs.*

**H3c.**  *Controlled motivation mediates the relationship between OCS and KTB among PhDs.*

### 2.5. Mediating Role of Amotivation

SDT defines amotivation as the absence of intention to act because the activity is not valued, is perceived as beyond one’s capability, or is not expected to affect outcomes (Deci & Ryan, 2000). When intention is absent, people either do not initiate effortful behavior or perform it perfunctorily and without care, a pattern that is typically accompanied by low perceived competence and low perceived relevance (Ferger & Rechberg, 2024). For PhDs, the skills they value often diverge from those expected by industry employers (De Grande et al., 2014; Muurlink et al., 2024). OCS may magnify this divergence through status ambiguity, limited autonomy, and weak recognition of expertise, which erode confidence and the perceived meaningfulness of one’s contribution (Skakni et al., 2022). Under these conditions, PhDs may not see reasons to engage in knowledge transfer or may doubt that their input will be valued or efficacious, which increases amotivation (Howard et al., 2016).

Amotivation is negatively related to KTB because it undermines goal-directed persistence required for articulating knowledge, adapting content to recipients, and following through in exchanges (Howard et al., 2016). Individuals experiencing amotivation also report lower perceived competence, which further suppresses the impulse to contribute because they do not expect their input to make a difference (Legault et al., 2007). Evidence from higher education shows that amotivation predicts lower perceived knowledge transferability (Wang et al., 2020). In workplace settings, amotivation has been associated with inattentiveness and reduced diligence (Van den Broeck et al., 2021), which conflict with the attentive, iterative interaction required for high-quality knowledge transfer (Argote & Fahrenkopf, 2016). In addition, when individuals do not perceive their knowledge as valuable or relevant, they are unlikely to share it. Non-sharing may reflect an absence of perceived value rather than strategic hoarding, especially when individuals cannot see the purpose of disclosure (Rechberg, 2018). Accordingly, we hypothesize:

**H4a.**  *OCS is positively associated with amotivation among PhDs.*

**H4b.**  *Amotivation is negatively associated with KTB among PhDs.*

**H4c.**  *Amotivation mediates the relationship between OCS and KTB among PhDs.*

### 2.6. Individual Characteristics and Experiences as Exploratory Boundary Conditions

Both situational and individual factors shape employees’ KTB. However, empirical evidence remains limited on whether these influences vary across individuals. Engineering and technology fields are traditionally male-dominated (K. Li et al., 2025), and women in these settings often feel pressure to conform to masculine professional norms (Ingram, 2006). For female PhDs entering industry, this adaptation can be especially challenging because conventional organizational socialization processes rarely account for gender-specific responses (Kowtha, 2008). Empirical studies have revealed consistent gender gaps in KTB. Female scientists are less likely than their male peers to commercialize research results or to file patents and disclose inventions, and they tend to achieve lower technological impact (Abreu & Grinevich, 2017; Colyvas et al., 2012; Sugimoto et al., 2015). Such disparities have been linked to differences in risk preferences. Men generally exhibit lower risk aversion, which makes them more likely to pursue risky and uncertain KTB (Croson & Gneezy, 2009). Additionally, large-scale evaluations indicate that gender biases in recognizing and rewarding knowledge transfer achievements reduce the expected returns of these behaviors for women (Bustelo & Salido, 2024). These structural and motivational differences suggest that female PhDs may depend more on perceived organizational support and equitable recognition to engage in knowledge transfer, whereas male PhDs’ alignment with established norms and lower risk sensitivity allow them to internalize transfer-related goals even under constraint. Therefore, gender may serve as a meaningful boundary condition, shaping how OCS influences motivational dynamics and KTB among PhDs.

Career goal at PhD entry (e.g., academic vs. industry), prior industry collaboration experience, and dissertation orientation (e.g., applied vs. basic research) are conceptually interrelated and often co-occur as an industry-oriented profile. However, each factor captures a distinct facet of the doctoral experience, namely, the initial career intent, experiential exposure, and research focus, respectively. PhDs who began their doctoral studies with an industry-oriented career goal tend to be less susceptible to OCS’s demotivating effects and are better able to translate their motivation into KTB, as their expectations align with industry norms and practical applications (Kim et al., 2018; Roach & Sauermann, 2017). Similarly, prior industry collaboration during doctoral training can buffer OCS’s adverse impact on motivation while also enabling such PhDs to leverage their established industry relationships for knowledge transfer (Boman et al., 2026). Likewise, for PhDs who complete an applied research dissertation, the translational focus of their research may reduce the shock of transitioning into an industrial environment and help them channel their motivation into KTB (Conti & Visentin, 2015). Taken together, prior research suggests that gender, career goal at PhD entry, prior industry collaboration experience, and dissertation orientation may shape how newly hired PhDs experience organizational transition and translate motivation into knowledge transfer. However, existing evidence does not provide sufficiently precise grounds for predicting which specific structural paths should differ across each subgroup. Therefore, consistent with RQ3, we treat these variables as theoretically informed exploratory boundary conditions rather than as path-specific confirmatory hypotheses.

## 3. Methodology

### 3.1. Participants and Procedure

Culture shock is an active process of adaptation that typically occurs within the first year after a cross-cultural or cross-organizational transition (Ward et al., 2005). Therefore, the participants in this study were engineering PhD graduates who had been employed in industry for no more than one year. With the assistance of the Career Development Center at A University in Beijing, we designed and administered an online questionnaire survey embedded in the Professional Development Module of the university’s 2024 Doctoral Graduates Survey. The survey targeted doctoral graduates newly employed in firms, and this dataset was analyzed for the first time in the present manuscript. The survey link was distributed by email to eligible PhD graduates. On the instruction page of the questionnaire, we described the study’s objectives and significance and emphasized the voluntary, anonymous, and confidential nature of participation.

A total of 571 eligible PhD graduates were contacted by email, and 497 completed the survey. To ensure the quality of the data, we removed 31 responses that exhibited straight-line response patterns (Zhang & Conrad, 2014). This resulted in 466 valid responses, which were used for the subsequent data analysis. Chi-square tests revealed no significant demographic differences between the final sample and the original sample (*p* > 0.05). The background information for the final sample is presented in Table 1.

### 3.2. Measurement

The questionnaire comprised two sections. In the first section, we collected respondents’ background information, including gender (1 = male, 0 = female), career goal at PhD entry (1 = industry, 0 = other), industry collaboration experience (1 = yes, 0 = no), and dissertation orientation (1 = applied, 0 = other). These variables were operationalized as binary indicators. In the second section, participants completed scales assessing the five focal constructs in this study. Because the sample consisted of Chinese PhD graduates, all measures originally developed in English were translated into Chinese using a translation/back-translation procedure (Schaffer & Riordan, 2003). All items were answered on a five-point Likert scale ranging from 1 (strongly disagree) to 5 (strongly agree). To ensure contextual appropriateness, item wordings were adapted to reflect the work roles and transition experiences of newly hired PhD employees in firms, while preserving the core conceptual meaning of original constructs. All items are presented in Appendix A.

Consistent with the application-focused definition introduced above, KTB was initially assessed with five items adapted and extended from prior studies (Ko et al., 2005; Tho & Trang, 2015; Tho, 2017). Following Tho (2017), KTB involves both the internalization of acquired knowledge and its application to work-related tasks and performance. Since the respondents in this study had already completed their doctoral training and entered firms, the present study focused on the application aspect of KTB. Specifically, the items capture the extent to which newly hired PhDs use the knowledge, skills, methods, techniques, and analytical capabilities developed during doctoral training in their current work. Based on conceptual alignment and psychometric evaluation, the final KTB measure retained the three items that most directly captured the use of doctoral-acquired expertise in current work. These items assess whether such expertise helps respondents enhance current job performance, apply specialized knowledge and research methods in professional practice, and solve complex problems in their current jobs.

Organizational culture shock was measured with a 10-item scale developed for this study based on the 10 salient challenges identified in a prior study of PhDs entering non-academic workplaces (Skakni et al., 2022). These items were designed to capture the main difficulties doctoral graduates may experience when they move from academic training into firms, including adjustment to daily workplace functioning, organizational values, and status hierarchies.

Autonomous motivation, controlled motivation, and amotivation were assessed using the Multidimensional Work Motivation scale (Gagné et al., 2015), with item wording adapted to the context of knowledge transfer at work. Specifically, autonomous motivation comprised five items and assessed the extent to which PhDs engaged in knowledge transfer out of personal interest, enjoyment, and perceived value. Controlled motivation also comprised five items and captured the extent to which PhDs engaged in knowledge transfer because of external expectations, approval, rewards, or internal pressure. Amotivation comprised three items and measured the extent to which PhDs lacked clear intention, value, or purpose in engaging in knowledge transfer at work.

### 3.3. Data Analysis

The data analysis proceeded in two main stages recommended by Anderson and Gerbing (1988). First, we evaluated the measurement quality of the latent constructs before estimating the substantive relationships in the proposed model. Specifically, confirmatory factor analyses (CFAs) were conducted for the five-factor measurement model to examine item loadings, internal consistency, convergent validity, and discriminant validity. Because all focal variables were obtained from the same questionnaire survey, we examined the possibility of common method bias by adding an unmeasured common latent factor to the CFA model and comparing the resulting model with the baseline measurement model (Podsakoff et al., 2003). Second, we estimated a latent-variable structural equation model (SEM) to assess the direct and indirect associations among OCS, the three SDT motivation variables, and KTB.

Model fit was assessed using multiple fit statistics. The criteria included the ratio of chi-square to the degrees of freedom (*χ*^2^/*df*) below 5, comparative fit index (CFI) and Tucker–Lewis index (TLI) values above 0.90, standardized root mean square residual (SRMR) values below 0.08, and root mean square error of approximation (RMSEA) values below 0.08 (Kline, 2011). For the mediation analysis, we used a bootstrapping procedure with 5000 resamples to generate bias-corrected estimates of the indirect effects and their two-tailed 95% confidence intervals (CIs). An indirect effect was considered statistically significant when the corresponding 95% CI did not include zero (Preacher & Hayes, 2008). Finally, to address the exploratory boundary-condition question, we conducted multi-group SEM analyses to explore whether the structural relationships differed across subgroups defined by gender, career goal at PhD entry, industry collaboration experience, and dissertation orientation. All statistical analyses were implemented in Amos 26.

## 4. Results

### 4.1. Measurement Model

Before conducting CFA, we examined the skewness and kurtosis of measurement items. As shown in Table 2, the absolute values of skewness of items were between 0.054 and 0.940, which are below the threshold of 3, and the absolute values of kurtosis of items ranged from 0.020 and 1.988, which are below the cutoff of 10. These results indicated approximately normal distributions and the data were suitable for subsequent SEM analyses (Kline, 2011).

We then conducted a series of CFAs to test the fit of the proposed five-factor measurement model and the distinctiveness of the focal variables. The model specified five latent variables and 26 observed indicators, with latent constructs freely correlated and each indicator constrained to load on its corresponding construct. The results indicated that the measurement model exhibited a good fit to the data (*χ*^2^ = 587.340; *df* = 289; *χ*^2^/*df* = 2.032; CFI = 0.957; TLI = 0.951; SRMR = 0.039; RMSEA = 0.047 [90% CI: 0.042, 0.053]). As reported in Table 2, all standardized factor loadings were significant and exceeded the recommended value of 0.50 (Kline, 2011).

We calculated Cronbach’s α, composite reliability (CR), the average variance extracted (AVE), and inter-construct correlations. As shown in Table 2, Cronbach’s α coefficients ranged from 0.744 to 0.916, which surpassed the 0.700 benchmark (Nunnally, 1978). CR values ranged from 0.747 to 0.918, which also exceeded the 0.700 threshold. AVE values ranged from 0.497 to 0.737. The AVE of KTB was 0.497, which was marginally below the recommended threshold of 0.50. Given that this value was very close to the threshold, the composite reliability was acceptable, and the retained items were theoretically aligned with the application-focused definition of KTB; we retained the refined KTB measure in the final analyses. Nevertheless, this marginally below-threshold AVE suggests that the convergent validity and measurement precision of KTB should be interpreted with caution. As presented in Table 3, the square roots of the AVE for each construct were greater than the corresponding inter-construct correlation coefficients. These findings indicate that the measurement model demonstrated satisfactory convergent and discriminant validity (Fornell & Larcker, 1981). Furthermore, the correlations among OCS, the three SDT motivation mediators, and KTB were significant and aligned with theoretical expectations.

We further compared the hypothesized five-factor model with four alternative models that combined the constructs into one to four factors. As indicated in Table 4, none of the alternative models achieved acceptable fit, which provides additional evidence for discriminant validity among the five focal constructs. Among these alternative models, the one-factor CFA model showed particularly poor fit to the data, suggesting that the covariance among the measurement items was unlikely to be adequately represented by one general factor. We also evaluated potential common method bias by using the CFA-based common latent factor approach. Specifically, an unmeasured common latent factor was added to the hypothesized five-factor model to capture the shared variance among all indicators beyond their substantive constructs. Although the six-factor model improved the *χ*^2^ value relative to the five-factor model (Δ*χ*^2^ [Δ*df* = 26] = 125.926, *p* < 0.001), the changes in CFI (ΔCFI = 0.014) and RMSEA (ΔRMSEA = 0.007) did not exceed the 0.05 rule of thumb (Bagozzi & Yi, 1990). Thus, these results suggest that common method bias was unlikely to be the dominant explanation for the observed relationships. Additionally, the results of the multicollinearity test showed that all independent variables had a Tolerance value above 0.10 (ranged from 0.677 to 0.950) and a VIF (Variance Inflation Factor) value below 5 (ranged from 1.052 to 1.478), indicating no severe multicollinearity in this study (Kline, 2011).

### 4.2. Structural Model

**Figure 2:**
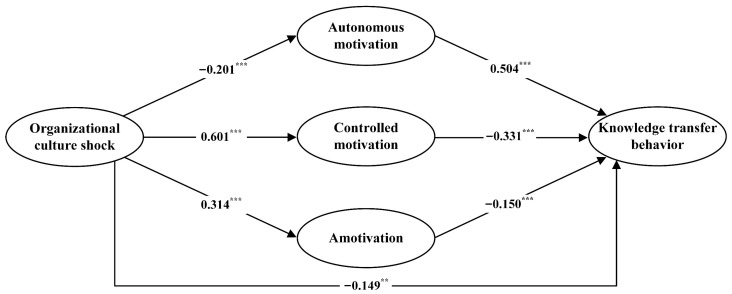
Results of the SEM analysis. Note: ** *p* < 0.01; *** *p* < 0.001.

We tested the hypothesized relationships among the focal variables using SEM. The structural model showed good fit to the data (*χ*^2^ = 603.956; *df* = 292; *χ*^2^/*df* = 2.068; CFI = 0.955; TLI = 0.950; SRMR = 0.047; RMSEA = 0.048 [90% CI: 0.043, 0.053]). Figure 2 displays the standardized path coefficients and their significance levels. Specifically, the direct association between OCS and KTB was negative and significant (*β* = −0.149, *SE* = 0.049, *p* < 0.01), supporting H1. As expected, OCS was negatively associated with autonomous motivation (*β* = −0.201, *SE* = 0.056, *p* < 0.001), and autonomous motivation was positively associated with KTB (*β* = 0.504, *SE* = 0.043, *p* < 0.001), supporting H2a and H2b. Bootstrap analysis indicated a significant negative indirect effect through autonomous motivation (indirect effect = −0.101, *SE* = 0.036, 95% CI [−0.180, −0.039]), supporting H2c. OCS was positively associated with controlled motivation (*β* = 0.601, *SE* = 0.071, *p* < 0.001), and controlled motivation was negatively related to KTB (*β* = −0.331, *SE* = 0.037, *p* < 0.001), supporting H3a and H3b. The negative indirect effect through controlled motivation was significant (indirect effect = −0.199, SE = 0.049, 95% CI [−0.297, −0.106]), supporting H3c. OCS was also positively related to amotivation (*β* = 0.314, *SE* = 0.053, *p* < 0.001), and amotivation was negatively related to KTB (*β* = −0.150, *SE* = 0.040, *p* < 0.001). The negative indirect effect through amotivation was also significant (indirect effect = −0.047, *SE* = 0.023, 95% CI [−0.102, −0.008]).

### 4.3. Exploratory Multi-Group Analysis of Boundary Conditions

To address RQ3, we conducted a series of multi-group SEM analyses to examine whether the structural relationships among OCS, SDT motivations, and KTB differed across subgroups defined by gender, career goal at PhD entry, industry collaboration experience, and dissertation orientation. Because we did not specify path-level confirmatory hypotheses for each moderator, these analyses are interpreted as exploratory tests of boundary conditions. Specifically, we compared an unconstrained model (structural paths were freely estimated across groups) with a constrained model (structural paths were set equal). To ensure measurement equivalence, factor loadings were held invariant across groups and residuals were freely estimated in both models. A significant *χ*^2^ difference between the two models indicated a moderating effect (Wu, 2013). As shown in Table 5, *χ*^2^ difference tests were significant for all four moderators, which suggested subgroup variation by gender, career goal at PhD entry, industry collaboration experience, and dissertation orientation.

To identify which paths differed across groups, we followed Augustus-Horvath and Tylka (2009) and compared each constrained model with seven partially unconstrained models that freed one target path at a time. As shown in Table 6, gender moderated two of the seven paths. Specifically, the positive association between autonomous motivation and KTB was weaker for female PhDs (*β* = 0.358, *SE* = 0.057, *p* < 0.001) than for male PhDs (*β* = 0.571, *SE* = 0.051, *p* < 0.001), whereas the negative association between controlled motivation and KTB was stronger for female PhDs (*β* = −0.460, *SE* = 0.052, *p* < 0.001) than for male PhDs (*β* = −0.190, *SE* = 0.041, *p* < 0.01).

In terms of career goal at PhD entry, the negative association between controlled motivation and KTB was weaker for the industry-goal group (*β* = −0.260, *SE* = 0.043, *p* < 0.01) than for the other-goal group (*β* = −0.363, *SE* = 0.049, *p* < 0.001). Similarly, the negative association between amotivation and KTB was weaker for the industry-goal group (*β* = −0.021, *SE* = 0.060, *p* > 0.05) than for the other-goal group (*β* = −0.265, *SE* = 0.052, *p* < 0.001).

Regarding industry collaboration experience, the negative associations of OCS with autonomous motivation and KTB were weaker among PhDs with collaboration experience (for autonomous motivation: *β* = −0.080, *SE* = 0.069, *p* > 0.05; for KTB: *β* = −0.031, *SE* = 0.060, *p* > 0.05) than among those without such experience (for autonomous motivation: *β* = −0.353, *SE* = 0.086, *p* < 0.001; for KTB: *β* = −0.322, *SE* = 0.064, *p* < 0.001); the positive association between OCS and controlled motivation was weaker for those with collaboration experience (*β* = 0.583, *SE* = 0.080, *p* < 0.001) than for those without collaboration experience (*β* = 0.633, *SE* = 0.101, *p* < 0.001); the positive association between autonomous motivation and KTB was stronger for those with collaboration experience (*β* = 0.627, *SE* = 0.056, *p* < 0.001) than for those without collaboration experience (*β* = 0.350, *SE* = 0.055, *p* < 0.001).

Finally, dissertation orientation moderated two paths. The negative association between OCS and autonomous motivation was weaker for applied-dissertation PhDs (*β* = −0.107, *SE* = 0.066, *p* > 0.05) than for those with other orientations (*β* = −0.409, *SE* = 0.095, *p* < 0.001); the negative association between amotivation and KTB was also weaker for the applied-dissertation group (*β* = −0.054, *SE* = 0.047, *p* > 0.05) than for the other group (*β* = −0.310, *SE* = 0.064, *p* < 0.001). Overall, these exploratory multi-group results indicate that the OCS–motivation–KTB process varies across several individual and experiential subgroups. Because these analyses were not based on path-specific confirmatory hypotheses, the significant path differences are interpreted as descriptive evidence of subgroup variation.

## 5. Discussion and Implications

Recognizing that newly hired engineering PhDs can serve as a crucial channel of knowledge transfer from universities to firms, this study examined how OCS is associated with their KTB in firms. Drawing on SDT, we examined whether OCS was directly and indirectly associated with KTB through SDT motivations and explored whether these associations varied by gender, career goal at PhD entry, prior industry collaboration experience, and dissertation orientation. Using survey data from engineering PhDs within one year of entering industry, we found that higher OCS was associated with lower KTB, both directly and indirectly through lower autonomous motivation, higher controlled motivation, and higher amotivation. The strength of these associations also differed across the four subgroups. Overall, this study extends research on PhDs in industry from employment outcomes to actual workplace behavior, and it extends SDT-based doctoral career research from career preferences to post-graduation motivation and knowledge transfer in firms.

### 5.1. Theoretical Interpretation: Organizational Culture Shock as a Socialization and Boundary-Transition Problem

First, the negative direct association between OCS and KTB remained significant after accounting for motivational mechanisms. Newly hired PhDs who perceived a stronger misfit in routines, values and status hierarchies reported lower levels of applying and disseminating their doctoral knowledge at work. This finding is consistent with prior studies showing that cultural distance between the knowledge source and recipient is linked to higher coordination and translation costs in knowledge transfer (Fong Boh et al., 2013; Rahman et al., 2018). It also echoes research on PhDs’ transition from academia to industry, where differences in organizational structures and knowledge-production logics are often salient (Leydesdorff & Meyer, 2006; Skakni et al., 2022).

OCS should therefore be interpreted not only as newcomers’ psychological discomfort or individual failure to adapt, but as a socialization and boundary-transition problem. During the transition from academia to industry, PhDs need to renegotiate their professional identity, role legitimacy, and ways of demonstrating value. Lower KTB may thus reflect not only weaker willingness to transfer knowledge, but also unclear role expectations, insufficient recognition of doctoral expertise, and limited organizational arrangements for translating academic knowledge into firm-specific practices. This interpretation connects the literature on PhDs’ career transitions with research on cross-boundary knowledge transfer and scientific human capital (Bozeman & Mangematin, 2004; Hernando, 2015).

Second, our findings on the SDT motivational pathways were largely in line with prior research. OCS was negatively associated with autonomous motivation and positively associated with controlled motivation and amotivation. In turn, autonomous motivation was positively associated with KTB, whereas controlled motivation and amotivation were negatively associated with KTB. These findings support the core proposition of SDT that contextual conditions shape the quality, not only the level, of motivation by supporting or thwarting basic needs for autonomy, competence and relatedness (Deci & Ryan, 2000; Ryan & Deci, 2017). They also resonate with studies showing that autonomy-supportive contexts foster internalization and volitional engagement, whereas controlling contexts foster compliance and disengagement (Gagné, 2009; Gagné & Deci, 2005; Van den Broeck et al., 2021).

These findings also clarify the motivational meaning of knowledge transfer. Firms may not fully benefit from PhDs’ expertise when knowledge transfer is driven mainly by external pressure, evaluation concerns, or weakly internalized goals. Autonomous motivation appears to be linked to more voluntary, meaningful, and self-endorsed knowledge exchange (Reinholt et al., 2011; Argote, 2024). By contrast, controlled motivation may be associated with more compliance-oriented transfer, such as meeting immediate evaluation demands without engaging deeply in explanation, adaptation, or collaborative problem solving. This interpretation is in line with arguments that external pressure can lead to shallow and transactional exchanges (e.g., Minbaeva & Santangelo, 2018), whereas amotivation undermines persistent, goal-directed knowledge behaviors (e.g., Ferger & Rechberg, 2024). Thus, this study contributes by showing that OCS is not simply related to the amount of motivation; it is also related to the type of motivation through which PhDs engage in KTB. At the same time, because the AVE for KTB remained marginally below the conventional 0.50 threshold, the precision of pathway estimates involving KTB should be interpreted with some residual measurement uncertainty, even though the refined measure showed acceptable reliability and theoretical alignment with the application-focused construct definition.

Third, the multi-group analyses show that these motivational pathways are contingent on individual characteristics and prior experiences. Industry-oriented career goals, prior industry collaboration, and applied dissertation orientation may serve as pre-entry socialization resources because they expose PhDs to industrial norms, applied problem framing, and the language of practical use before organizational entry. This interpretation is consistent with research showing that industry exposure and applied research experiences may improve the alignment between doctoral training and non-academic work (Boman et al., 2026; Conti & Visentin, 2015; Roach & Sauermann, 2017). In this sense, our findings link doctoral education more directly to organizational socialization. Prior industry exposure and applied research experiences do not simply predict where PhDs work; they also shape how effectively PhDs can cross the academia–industry boundary after entry.

At the same time, the gendered pattern should be interpreted as descriptive rather than mechanistic. The results indicate that the motivation–KTB association differs by gender, but they do not show that female PhDs are less willing or less capable of transferring knowledge. One possible explanation is that women in male-dominated STEM workplaces may face greater scrutiny or receive less recognition for knowledge-related contributions, which may make autonomous motivation less easily converted into visible KTB and make externally pressured motivation more detrimental (Bustelo & Salido, 2024). However, other mechanisms, such as gender differences in workplace network position, access to mentorship, task assignments, supervisor support, or perceptions of organizational climate, may also account for this pattern.

### 5.2. Practical Implications for Firms and Doctoral Programs

For firms, the findings suggest that recruiting PhDs is not enough to ensure knowledge transfer. Doctoral expertise may remain underused if newcomers face unclear roles, unfamiliar routines, or weak recognition of their expertise. Firms may therefore consider transition practices that make doctoral knowledge visible and usable. For example, realistic job previews can clarify how industrial research differs from academic inquiry, what outputs are valued, and how doctoral expertise may contribute to projects. During onboarding, supervisors and technical mentors can help PhD newcomers map their research expertise onto concrete organizational problems, thus facilitating newcomer adjustment and socialization (Lee, 2024).

The negative association between controlled motivation and KTB also has implications for performance management. Firms may wish to be cautious about relying mainly on short-term KPIs, monetary incentives, or pressure-based evaluation to elicit knowledge transfer from PhDs. Such mechanisms may encourage visible compliance, but they may be less conducive to deeper forms of knowledge transfer, such as adapting academic methods to industrial constraints, diagnosing complex technical problems, and engaging in collaborative problem solving. Evaluation systems may therefore need to recognize both tangible outputs and process-oriented contributions, including mentoring colleagues, translating research methods into usable tools, and building bridges with external scientific networks.

Finally, the exploratory subgroup findings suggest that a one-size-fits-all approach may be insufficient. PhDs without prior industry collaboration experience or with less application-oriented research backgrounds may need more explicit support in understanding industrial problem framing, project timelines, and the expected balance between scientific rigor and practical delivery (Skakni et al., 2022). Those who did not originally plan to enter industry may benefit from support in reconstructing their professional identity and social networks (Hancock & Walsh, 2016). For female PhDs, the observed gender differences suggest that firms may need to examine whether female and male PhDs have equitable access to high-value projects, mentorship, recognition, and psychologically safe team climates.

For doctoral programs, the preparation of PhD graduates for industry should go beyond career information. Programs may provide industry-facing research projects, short-term placements, or co-supervised projects with firm partners, which are consistent with prior work on collaborative doctoral education and knowledge mediation (Liu et al., 2020). Career development modules could also include training in knowledge translation, such as how to convert research methods, analytical models, experimental routines, or dissertation findings into tools that non-academic colleagues can understand and use.

## 6. Limitations and Future Research Directions

Several limitations warrant consideration in future research. First, the cross-sectional and single-source survey design precludes strong causal inference and raises method-related concerns. Although the proposed order of OCS, motivation and KTB is grounded in SDT and in longitudinal research on need-supportive work climates (Gagné & Deci, 2005; Van den Broeck et al., 2021), alternative causal directions cannot be fully ruled out. For example, successful application of doctoral-acquired knowledge may strengthen autonomous motivation and reduce controlled motivation or amotivation over time, suggesting a possible KTB-to-motivation pathway. It is also possible that both SDT motivations and KTB are jointly shaped by person–job fit, or by the alignment between doctoral training, doctoral-acquired expertise, and current job content. Because the present survey did not include an independent validated measure of person–job fit or PhD–job fit, this alternative explanation could not be tested directly. Future longitudinal studies or experimental designs should examine reciprocal relationships among OCS, SDT motivations, and KTB while measuring PhD–job fit separately. In addition, future work could combine self-reports with supervisor ratings, objective indicators of knowledge outputs, or network-based measures of knowledge flows (Argote & Fahrenkopf, 2016; Thomas & Gupta, 2022).

Second, the generalizability of the findings should be interpreted with caution. Our sample consisted of engineering PhDs working in Chinese firms within one year after graduation. Moreover, respondents were recruited through a single university-based administrative channel and shared a common institutional training environment. The sample is therefore not a representative sample of all engineering PhDs in China or all PhDs entering firms. This specific group may limit the extent to which the findings can be applied to other disciplines, career stages or national contexts. Accordingly, the implications of this study are most directly relevant to newly hired engineering PhDs from similar Chinese doctoral training contexts who enter firm settings. Future comparative studies across different countries, sectors, and cohorts would help clarify the boundary conditions of the model.

Third, the measurement of KTB should be further developed. Our KTB scale captures the extent to which newly hired PhDs use doctoral-acquired knowledge, skills, methods, and analytical capabilities in current work. However, it does not fully capture broader transfer processes, such as interpersonal knowledge sharing or knowledge translation across actors, or the organizational uptake of doctoral expertise. Future research could develop and validate more fine-grained KTB measures that distinguish among knowledge application, sharing, translation, and integration. Such measures would provide a more detailed understanding of how doctoral expertise moves from individual knowledge to organizational use.

Finally, the present model focuses on SDT motivations and selected individual characteristics. For example, the exploratory gender findings indicate that the motivation–KTB association varies by gender, but they do not identify the mechanisms behind this difference. Future studies should examine whether workplace network position, access to mentorship, task assignments, supervisor support, recognition, or team climate explains these patterns. More broadly, contextual factors such as autonomy-supportive supervision, team learning climate, and formal knowledge management practices may interact with OCS and motivation in shaping KTB. Individual resources, such as proactive personality or psychological capital, may also matter (Haivas et al., 2014; Thomas & Gupta, 2022). Exploring these organizational and individual conditions would deepen our understanding of when newly hired PhDs are more likely to translate doctoral expertise into organizationally useful knowledge.

## Figures and Tables

**Table 1:** Background characteristics of the PhD respondents (*N* = 466).

Variable	Group	*n*	Percentage (%)
Gender	Female	181	38.84
	Male	285	61.16
Career goal at PhD entry	Industry	229	49.14
	Other	237	50.86
Industry collaboration experience	Yes	227	48.71
	No	239	51.29
Dissertation orientation	Applied	324	69.53
	Other	142	30.47

**Table 2:** Descriptive statistics, factor loadings, reliability and validity of the variables.

Variables	Items	Skewness	Kurtosis	Loadings	α	CR	AVE
knowledge transfer behavior (KTB)	KTB1	−0.940	1.988	0.759	0.744	0.747	0.497
	KTB2	−0.861	0.789	0.642			
	KTB3	−0.748	1.010	0.710			
Organizational culture shock (OCS)	OCS1	0.286	−0.226	0.720	0.916	0.912	0.511
	OCS2	0.450	−0.088	0.673			
	OCS3	0.365	−0.396	0.767			
	OCS4	0.416	−0.434	0.713			
	OCS5	0.435	−0.090	0.794			
	OCS6	0.479	−0.321	0.700			
	OCS7	0.611	0.020	0.745			
	OCS8	0.333	−0.336	0.682			
	OCS9	0.279	−0.462	0.688			
	OCS10	0.438	−0.217	0.757			
Autonomous motivation (AUM)	AUM1	−0.513	−0.206	0.739	0.870	0.873	0.579
	AUM2	−0.621	0.141	0.802			
	AUM3	−0.530	−0.206	0.771			
	AUM4	−0.582	−0.228	0.813			
	AUM5	−0.672	0.129	0.671			
Controlled motivation (COM)	COM1	0.444	−0.597	0.763	0.916	0.918	0.693
	COM2	0.195	−0.489	0.833			
	COM3	0.539	−0.202	0.816			
	COM4	0.233	−0.515	0.879			
	COM5	0.305	−0.470	0.865			
Amotivation (AMO)	AMO1	0.054	0.064	0.916	0.888	0.893	0.737
	AMO2	0.119	0.052	0.877			
	AMO3	0.271	0.193	0.776			

**Table 3:** Means, standard deviations, and correlations of variables.

Variables	1	2	3	4	5	6	7	8	9
1	—								
2	−0.457 ***	—							
3	0.001	0.142 **	—						
4	−0.040	0.049	0.095 *	—					
5	0.061	−0.020	0.106 *	0.048	***0.715***				
6	0.120 ***	−0.029	0.126 **	−0.064	−0.179 ***	***0.761***			
7	−0.045	0.060	0.027	0.013	0.548 ***	−0.170 ***	***0.832***		
8	0.260 ***	−0.231 ***	−0.116 *	−0.120 **	0.275 ***	−0.157 ***	0.266 ***	***0.858***	
9	0.118 *	−0.075	0.031	0.016	−0.398 ***	0.495 ***	−0.454 ***	−0.278 ***	***0.705***
M	0.612	0.491	0.487	0.695	2.419	3.754	2.518	2.431	3.793
SD	0.488	0.500	0.500	0.461	0.729	0.769	0.906	0.746	0.663

Note: 1 = gender; 2 = career goal at PhD entry; 3 = industry collaboration experience; 4 = dissertation orientation; 5 = organizational culture shock; 6 = autonomous motivation; 7 = controlled motivation; 8 = amotivation; 9 = knowledge transfer behavior. The diagonal elements (in bold italics) indicate the square roots of the AVE values. * *p* < 0.05; ** *p* < 0.01; *** *p* < 0.001.

**Table 4:** Results of the model comparisons.

Models	*χ*^2^ (*df*)	*χ*^2^/*df*	CFI	RMSEA
One-factor (abcde)	3612.995 (299)	12.084	0.521	0.154
Two-factor (a-bcde)	2690.126 (298)	9.027	0.654	0.131
Three-factor (a-bcd-e)	2506.156 (296)	8.467	0.680	0.127
Four-factor (a-bc-d-e)	1712.132 (293)	5.843	0.795	0.102
Five-factor (a-b-c-d-e)	587.340 (289)	2.032	0.957	0.047
Six-factor (a-b-c-d-e-f)	461.414 (263)	1.754	0.971	0.040

Note: a = organizational culture shock; b = autonomous motivation; c = controlled motivation; d = amotivation; e = knowledge transfer behavior; f = common method factor.

**Table 5:** Chi-square difference between the constrained model and the unconstrained model.

Moderators	Model	*χ*^2^ (*df*)	*χ*^2^/*df*	Δ*χ*^2^ (∆*df*)
Gender	Unconstrained	1073.742 (605)	1.775	31.822 *** (7)
	Constrained	1105.564 (612)	1.806	
Career goal at PhD entry	Unconstrained	980.826 (605)	1.621	17.786 * (7)
	Constrained	998.612 (612)	1.632	
Industry collaboration experience	Unconstrained	1013.223 (605)	1.675	35.967 *** (7)
	Constrained	1049.189 (612)	1.714	
Dissertation orientation	Unconstrained	1074.049 (605)	1.775	16.577 * (7)
	Constrained	1090.626 (612)	1.782	

Note: * *p* < 0.05; *** *p* < 0.001.

**Table 6:** Results of the multi-group SEM analysis.

	Standardized Coefficients		*χ*^2^ (*df*)	Δ*χ*^2^ (Δ*df*)
**Gender**	**Female**	**Male**	—	—
**Constrained Model**	—	—	1105.564 (612)	—
AUM → KTB	0.358 ***	0.571 ***	1096.771 (611)	8.793 **
COM → KTB	−0.460 ***	−0.190 **	1091.097 (611)	14.467 ***
**Career goal at PhD entry**	**Industry**	**Other**	—	—
**Constrained Model**	—	—	998.612 (612)	—
COM → KTB	−0.260 ***	−0.363 ***	994.315 (611)	4.297 *
AMO → KTB	−0.021	−0.265 ***	990.950 (611)	7.662 *
**Industry collaboration experience**	**Yes**	**No**	—	—
**Constrained Model**	—	—	1049.189 (612)	—
OCS → KTB	−0.031	−0.322 ***	1036.447 (611)	12.742 ***
OCS → AUM	−0.080	−0.353 ***	1039.056 (611)	10.133 **
OCS → COM	0.583 ***	0.633 ***	1042.863 (611)	6.326 *
AUM → KTB	0.627 ***	0.350 ***	1040.989 (611)	8.200 **
**Dissertation orientation**	**Applied**	**Other**	—	—
**Constrained Model**	—	—	1090.626 (612)	—
OCS → AUM	−0.107	−0.409 ***	1082.668 (611)	7.958 **
AMO → KTB	−0.054	−0.310 ***	1082.747 (611)	7.879 **

Note: Only paths with significant differences are reported. OCS = organizational culture shock; AUM = autonomous motivation; COM = controlled motivation; AMO = amotivation; KTB = knowledge transfer behavior. * *p* < 0.05; ** *p* < 0.01; *** *p* < 0.001.

## Data Availability

The data presented in this study are available on request from the corresponding author due to privacy and confidentiality restrictions related to participant information.

## Appendix A. Questionnaires

### Appendix A.1. Knowledge Transfer Behavior (Ko et al., 2005; Tho & Trang, 2015; Tho, 2017; Items Excluded from the Final Analyses Are Marked with an Asterisk)

1. The knowledge and skills I acquired during my PhD help me enhance my current job performance.
2. I have successfully applied the specialized knowledge, research methods, or techniques I developed during my PhD to my professional practice.
3. The knowledge and skills I acquired during my PhD help me solve complex problems in my current job.
4. The knowledge and skills I acquired during my PhD are highly applicable to my current job. *
5. The knowledge and skills I acquired during my PhD are needed for my current job. *

### Appendix A.2. Organizational Culture Shock (Skakni et al., 2022)

1. I was surprised by the strict control over working hours and the limited flexibility in scheduling my tasks.
2. I found it hard to adapt to new ways of doing tasks.
3. I struggled to adjust to a highly collaborative work environment.
4. I was surprised by the lower standards of rigor and productivity compared to academia.
5. I found it hard to adapt to the results-oriented way of thinking in my current job.
6. I had to adjust my communication style to fit the way people interact at work.
7. My employer’s expectations felt higher than what I was used to in academia.
8. I found it unclear what my role and position were in the organization.
9. I had less decision-making freedom than I was used to during my PhD.
10. I felt my expertise wasn’t fully recognized or appreciated.

### Appendix A.3. Autonomous Motivation (Gagné et al., 2015)

1. I derive much pleasure from disseminating my knowledge.
2. I personally consider it important to disseminate knowledge in my work.
3. Disseminating my knowledge is personally significant to me.
4. Disseminating my knowledge aligns with my personal values.
5. I feel satisfied when I successfully disseminate knowledge in my work.

### Appendix A.4. Controlled Motivation (Gagné et al., 2015)

1. I disseminate knowledge in my work to gain others’ approval (e.g., supervisor, colleagues, family, clients).
2. I disseminate knowledge in my work because I have to prove to myself that I can.
3. I disseminate knowledge in my work because it makes me feel proud of myself.
4. I disseminate knowledge in my work because it offers me greater job security.
5. I disseminate knowledge in my work because it brings me material rewards.

### Appendix A.5. Amotivation (Gagné et al., 2015)

1. I do not always see a reason to disseminate knowledge in my work.
2. I do not always see the purpose of disseminating knowledge in my work.
3. I do not always see the relevance of disseminating knowledge in my work.
